# Recent Developments in Lateral Flow Assays for *Salmonella* Detection in Food Products: A Review

**DOI:** 10.3390/pathogens12121441

**Published:** 2023-12-13

**Authors:** Gabrielle B. L. Silva, Fabiana V. Campos, Marco C. C. Guimarães, Jairo P. Oliveira

**Affiliations:** Morphology Department, Health Sciences Center, Federal University of Espírito Santo, Av Marechal Campos 1468, Vitória 29040-090, Brazil; gabrielle.b.silva@edu.ufes.br (G.B.L.S.); fabiana.v.campos@ufes.br (F.V.C.); marco.guimaraes@ufes.br (M.C.C.G.)

**Keywords:** *Salmonella*, salmonellosis, lateral flow assay, nanomaterials

## Abstract

Salmonellosis is a disease transmitted by contaminated food and is one of the leading causes of infections worldwide, making the early detection of *Salmonella* of crucial importance for public health. However, current detection methods are laborious and time-consuming, thus impacting the entire food supply chain and leading to production losses and economic sanctions. To mitigate these issues, a number of different biosensors have been developed, including lateral flow assays (LFAs), which have emerged as valuable tools in pathogen detection due to their portability, ease of use, time efficiency, and cost effectiveness. The performance of LFAs has been considerably enhanced by the development of new nanomaterials over the years. In this review, we address the principles and formats of the assay and discuss future prospects and challenges with an emphasis on LFAs developed for the detection of different *Salmonella* serovars in food.

## 1. Introduction

According to the World Health Organization, *Salmonella* ranks among the most common pathogens associated with foodborne diseases worldwide [1]. Outbreaks of such diseases are primarily associated with the consumption of eggs and their derivatives, raw or undercooked meats, and contaminated water [2,3]. The public health issues caused by this microorganism have raised concerns among authorities and consumers alike, making its rapid and reliable detection of utmost importance for food quality monitoring in order to ensure the safety of consumers [4].

The conventional method for the identification of *Salmonella* in food carried out in laboratories relies on culture techniques, biochemical analysis, and serotyping. This approach, although thorough, is labor-intensive and time-consuming, often requiring over 48 h to yield results [5]. As an alternative, methods such as the enzyme-linked immunosorbent assay (ELISA) and molecular techniques like polymerase chain reaction (PCR) have been adopted for *Salmonella* detection. Although these methods offer improved specificity and sensitivity, reducing the analysis time by up to two days compared to the traditional approach, they are not without limitations. Notably, the time required for securing results remains substantial, and these methods also demand specialized equipment and skilled analysts [6,7].

Presently, lateral flow assays (LFAs) have seen an upsurge in their application for diagnostic purposes due to their ability to swiftly detect the presence of bacteria in food products [8], in addition to detecting a range of foodborne pathogens including *Salmonella* [9], *Listeria* [10], and *Escherichia coli* [11]. The LFA method has proven to be an effective, fast, and inexpensive detection technique. Results can be interpreted through visual inspection by the presence or absence of the test line, indicating positive and negative outcomes, respectively [12]. Over the past few years, research has led to significant advancements in LFA development, including novel signal enhancement techniques, the use of new markers such as gold nanoparticles (AuNPs) [13], carbon nanoparticles, quantum dots (QD), magnetic nanoparticles (MNPs) [14], simultaneous detection, and improved quantification systems.

In this review, we present the state-of-the-art use of LFAs for the detection of *Salmonella*. We highlight its classifications and serovars along with the traditional analysis methods still in use. Principles and formats of LFAs, markers, and bioreceptors are also discussed. Furthermore, we delve into the major advancements, challenges, and future prospects for the development of devices for this pathogen’s detection.

## 2. *Salmonella*

The *Salmonella* genus, composed of the rod-shaped, gram-negative bacteria species *Salmonella bongori* and *Salmonella enterica*, includes over 2600 serotypes that can cause diseases in humans [15]. Initially named by Lignières in 1900, this genus was first classified based on the host species and clinical symptoms. However, this method led to confusion as different serotypes from diverse hosts were assigned identical names, ignoring their unique etiological characteristics [16].

To address these inconsistencies, microbiologists Fritz Kauffman and Philip Bruce White overhauled the taxonomic system in 1920. Their efforts culminated in the Kauffman–White classification scheme, which relies on both classical and molecular methods to identify *Salmonella* serotypes by their specific antigens: “O,” “H,” and “Vi”. The “O” antigens, indicative of the bacterium’s virulence, are defined by the structure and composition of sugars in the outer membrane’s lipopolysaccharides. Most *Salmonella* possess “H” antigens, which are linked to the flagellum. The “Vi” antigen, however, is exclusive to encapsulated strains within the species. Together, these antigens play a critical role in the precise identification and classification of *Salmonella* species [17].

Infection can be triggered by the intake of water and/or food contaminated by this bacterium. Foods that are rich in moisture, protein, and carbohydrates, such as beef, pork, poultry, eggs, dairy products, seafood, and filled desserts, are particularly susceptible to spoilage [18]. Poultry meat and other types of meat are frequently associated with outbreaks. Salmonellosis associated with dairy products is typically due to either raw or improperly pasteurized milk, as well as cheese. As for egg-derived products, the ones most often implicated in outbreaks include egg-based salads, ice creams, and other homemade desserts. There are three distinct clinical manifestations of the disease: typhoid fever, enteric fever, and enteric infections. Symptoms vary among infected individuals and may range from gastroenteritis and vomiting to headaches and fever [19]. Treatment for these infections usually involves fluid intake and the administration of medications, including antibiotics.

## 3. *Salmonella* Detection Methods

**Figure 1:**
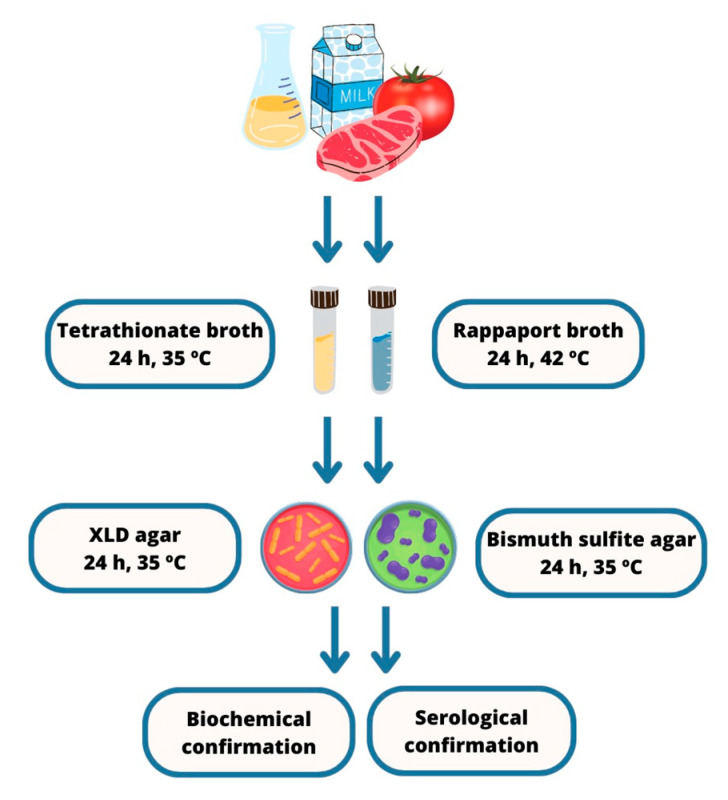
Isolation and confirmation steps according to the gold standard method. Adapted illustration [20].

The standard approach to *Salmonella* detection in food involves culturing and subculturing the bacteria on a variety of non-selective and selective culture media and incubating them at specific temperatures and timeframes. The traditional, widely adopted method ISO 6579:2002 [20] is considered the gold standard, comprising five consecutive steps (Figure 1): (a) Pre-enrichment in a non-selective broth to restore damaged cells to a stable physiological condition; (b) Selective enrichment, in which the sample is reintroduced into a culture broth containing inhibitory reagents that allow *Salmonella* growth while restricting that of most other bacteria; (c) Seeding onto selective solid media that inhibit the growth of non-*Salmonella* bacteria; (d) Biochemical tests to gather phenotypic data from isolated cultures; (e) Serotyping for antigenic characterization, marking the conclusive step in providing the specific identification of isolated cultures [21].

In addition to the conventional method, a few alternative techniques have been put into practice for *Salmonella* detection, including immunology-based methods such as the enzyme-linked immunosorbent assay (ELISA) and lateral flow immunochromatographic assays (LFAs). These techniques employ monoclonal or polyclonal antibodies that specifically bind to antigens present on the bacterium’s cell membrane [22]. These antibodies can detect somatic (O), flagellar (H) or capsular (Vi) antigens in a variety of food matrices [23,24].

Among the aforementioned assays, ELISA is the most widely used for the detection of *Salmonella*. In this method, when *Salmonella* antigens are present in a sample, they bind to anti-*Salmonella* antibodies that are immobilized on a solid matrix. The formation of this antigen–antibody complex is revealed through a color shift triggered by the enzymatic cleavage of a chromogenic substrate [25]. In 2019, di Febo et al. developed a capture ELISA test to detect *S*. *enterica* in various food matrices, having achieved similar results when compared to the official ISO method [26]. However, because of selectivity and sensitivity issues, for the detection of *Salmonella* and other pathogens in foodstuffs, the ELISA technique is usually enhanced by combining it with other methods [27]. Enhancements to ELISA include the use of fluorescent reporters [28], PCR amplification [29], and electrochemiluminescence [30] to boost signal detection. Notably, the use of AuNPs as chromogenic reporters has significantly increased the sensitivity of the ELISA, providing qualitative results indicated by color changes in the presence of the analyte [31]. For instance, an in situ immuno-AuNP network ELISA biosensor developed by Cho et al. in 2013 for the detection of *S*. Typhimurium and *E. coli* in liquid food samples successfully detected the presence of this *Salmonella* serotype in pineapple juice [32]. In yet another example of the use of nanomaterials to improve ELISA, carbon nanotubes conjugated to antibodies and horseradish peroxidase in both direct and sandwich forms of the assay decreased the limits of detection of *S*. Typhimurium in milk samples in up to 1000 cases [33].

Complementing these immunological methods are molecular-based assays that detect *Salmonella* through hybridization of DNA or RNA probes or primers to specific microbial sequences [34,35]. These methods include PCR, loop-mediated isothermal amplification (LAMP), nucleic acid sequence-based amplification (NASBA), and recombinase polymerase amplification (RPA).

PCR is renowned for its reliability and sensitivity, amplifying nucleic acids to identify *Salmonella* with precision [36]. Variations such as multiplex PCR [37] and real-time PCR (qPCR) [38] allow for simultaneous detection of multiple pathogens and quantitative analysis, respectively. Despite its efficacy, PCR can be inhibited by certain food components, like fats, which can interfere with the amplification process [39]. LAMP is an amplification technique that stands out for its efficiency and specificity under isothermal conditions [40], employing a unique DNA polymerase (Bst) and a set of four primers that can recognize six distinct target regions on DNA [41]. Liu et al., 2017 [41] developed multiplex-LAMP to detect *Salmonella* spp. and *Vibrio parahaemolyticus* with 100% specificity and Wu et al., 2015 [42] put forward an EMA-RTI-LAMP system to detect and quantify *S*. enteritidis. NASBA, yet another isothermal amplification method, targets RNA specifically through a transcription-based mechanism [43]. It offers high selectivity and overcomes some of the issues associated with PCR [44]. Its efficacy and sensitivity in detecting *Salmonella* have been well-documented [45]. Lastly, RPA is an advanced isothermal amplification method that rapidly amplifies as few as 1–10 DNA target copies within 20 min [46]. It has been employed to amplify single-stranded DNA, double-stranded DNA, RNA, and microRNA from a diverse range of samples and organisms [47]. RPA has been effectively employed for the detection of *Salmonella* in food samples [9].

Although the methods presented offer good sensitivity and specificity when it comes to identifying pathogenic bacteria, they are not without limitations. Some immunological assays, for instance, require prior sample enrichment to increase the bacterial count to detectable levels. Cross-reactivity with similar antigens from different *Salmonella* serovars or related bacteria, variations in antigenicity, sample matrix effects, and the costs of assay automation are also a challenge [22], not to mention laborious extraction and purification processes [48]. Moreover, effectiveness is highly dependent on the specific microbiota in the sample, the complexity of the food matrix, and any inhibitory substances present [24].

Although LFAs do not differentiate *Salmonella* serotypes, being employed only for triage purposes, these biosensors boast a range of advantages over the aforementioned methods, delivering swift results, good specificity, high sensitivity, and low detection limits. In addition, they are cost-effective to manufacture, user-friendly, and straightforward in terms of result interpretation, offering both qualitative and quantitative results [49,50,51].

## 4. Lateral Flow Assays (LFAs)

**Figure 2:**
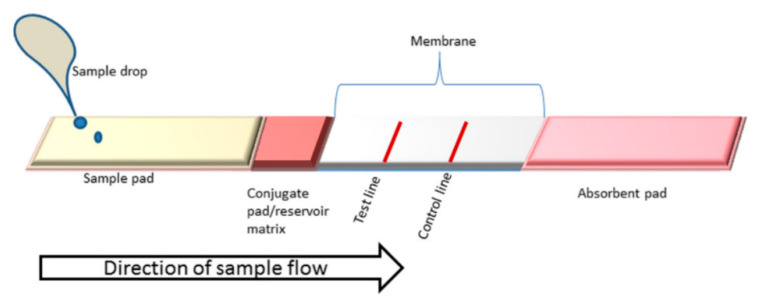
Components of an LFA strip. Illustration reprinted with permission from [56].

LFAs operate on the principle of immunochromatography, in which the sample flows through a solid substrate via capillary action. It is an assay designed to detect one or more analytes using bioreceptors such as aptamers, antigens, and antibodies, among others. These bioreceptors are conjugated with labels such as nanoparticles, liposomes, and enzymes to produce a signal that can manifest as fluorescent, colorimetric, or luminescent [52]. The LFA device comprises distinct components with specific functions: a sample pad, where the sample is applied for analysis; a conjugate pad, which contains immobilized bioreceptors and markers; a detection membrane, which holds the test and control lines; and an absorbent pad (Figure 2). The procedure for depositing conjugated particles onto the detection membrane can be performed either manually or automatically. The selection of the detection membrane is crucial for the manufacturing of LFA strips, as the adsorption of biomolecules is an essential factor for the device’s performance. Nitrocellulose membranes are the most used materials in point-of-care technologies [53], but, depending on the application, nylon and polyethersulfone membranes can also be used [54]. Another aspect to consider is the rate of capillary flow at which the sample advances and fills the detection surface, because flow time and test sensitivity are interconnected: the smaller the pore size, the more sensitive the test. As a result, the sample takes longer to cross the membrane, making the formation of an immunocomplex—comprised of the analyte and marker—more effective at the test line, where bioreceptors designed for analyte or complex capture are deposited. The test line displays qualitative or quantitative results, either by naked eye evaluation or with the assistance of equipment. Beyond the test line lies the control line, where additional capture bioreceptors are immobilized to confirm the validity of the device. The appearance of the control line validates the test; otherwise, the test is deemed invalid. After crossing the entire detection membrane, the sample is fully absorbed by the absorbent pad, thereby ensuring that the whole sample has traversed the membrane and does not backflow [55].

**Figure 3:**
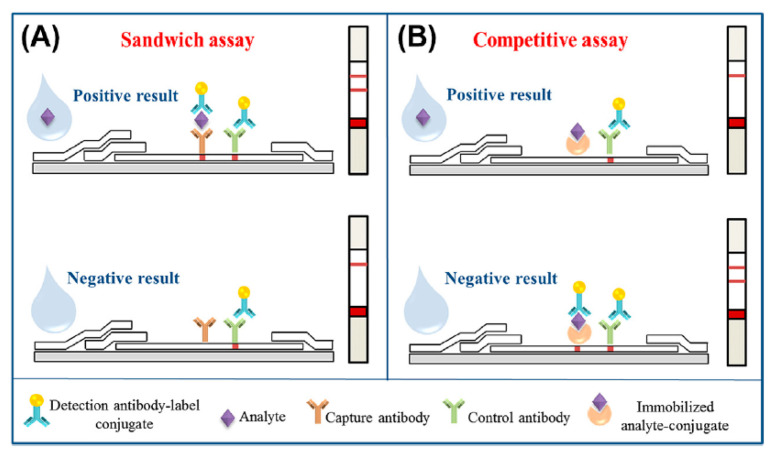
Lateral flow assay formats. Illustration reprinted with permission from [52].

There are two different formats of LFAs: the sandwich (direct) format and the competitive (indirect) one (Figure 3). The sandwich assay is used for the detection of high molecular weight molecules such as large proteins, which, much like *Salmonella*, present multiple antigenic sites. On the other hand, the competitive assay is used to detect low molecular weight molecules with a single antigen for antibody binding. In the sandwich format, if the analyte is present, both the test and control lines on the device will display color, whereas in the competitive format, the presence of the analyte is indicated by the absence of color on the test line, with only the control line being marked [57]. Regardless of the assay format selected, there must be a color display on the control line to confirm the validity of the test. Gao et al., 2021 [58] developed a sandwich LFA based on aptamer-magnetic separation and AuNPs for the detection of *Salmonella* Typhimurium, in which the detection limit achieved was 4.1 × 10^2^ CFU.mL^−1^ in real samples. In the competitive LFA developed by Chen et al., 2021 [59], different sizes of gold nanospheres were employed for the detection of mycotoxins in corn samples. This particular immunoassay exhibited accuracy and sensitivity in the results obtained.

### 4.1. Bioreceptors

Bioreceptors are vital components of LFAs for *Salmonella* detection. Essentially, any biomolecule capable of recognizing the analyte can serve as a bioreceptor [60]. Antibodies, aptamers, bacteriophages, and antimicrobial peptides (AMPs) feature among the most widely used bioreceptors for *Salmonella* detection [61].

Of those, antibodies stand out as the preferred choice due to their high specificity in binding to analytes. They possess a crystallizable fragment (Fc) region and an antigen-binding fragment for immune response and recognition [62]. In a study conducted by Lukman et al. in 2018 [63], a conjugate of AuNPs with antibodies was used for the detection of *S*. Typhi in water and milk samples, yielding a limit of detection (LOD) of 3 × 10^8^ CFU.mL^−1^. Hwang et al. in 2016 [64] used AuNPs and MNPs conjugated with antibodies to detect *Salmonella* in milk samples, reporting an LOD of 10^3^ CFU.mL^−1^.

Aptamers are single-stranded DNA or RNA obtained through the systematic evolution of ligands by exponential enrichment (SELEX) process. Their unique three-dimensional structures enable them to bind with high specificity and affinity to a wide array of targets that range from molecules of different molecular weights to whole cells [65]. Due to their physical and chemical stability, non-toxic nature, and ease of synthesis and modification, aptamers have become a significant tool in analyte recognition. These unique oligonucleotides can hybridize with their complementary DNA (cDNA) and undergo conformational changes in the presence of the target. Owing to these specific advantages, aptasensors have emerged as viable alternatives to antibodies in the detection of *Salmonella*. In 2015, Singh et al. [56] employed aptamers conjugated with AuNPs for the detection of *Salmonella* spp. They successfully identified 19 out of the 22 strains tested and achieved a detection limit in the range of 10^4^ to 10^6^ CFU.mL^−1^. In a study conducted by Bu et al. in 2020 [66], LFA strips were engineered using MNPs and aptamers for the detection of *Salmonella* Enteritidis, achieving an LOD between 10^2^ to 10^3^ CFU.mL^−1^.

**Figure 4:**
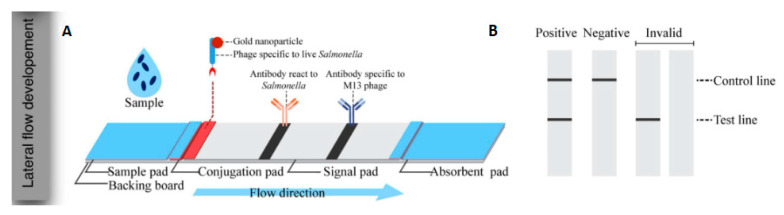
(**A**) Components of the LFA developed using AuNPs as a marker. (**B**) Illustration of the result reading on the LFA. Illustrations reprinted (adapted) with permission from [71].

Bacteriophages are viruses that infect bacteria and use their cells for replication cycles [67]. Biosensors developed from bacteriophages can differentiate between dead bacterial cells, as these viruses require a viable host to replicate. This property offers a distinct advantage over other types of biosensors [68]. A number of biosensors designed for *Salmonella* detection employ bacteriophages engineered to interact specifically with the target bacteria. This interaction prompts the bacteria to express luminescent enzymes, which allows the detection of foodborne pathogens with a degree of sensitivity that surpasses other detection methods such as immunoassays and PCR. However, one of the challenges of using bacteriophages as bioreceptors is the stability of their capture activity, which may be lost when they are dried out. Furthermore, the lysis of bacterial cells can occur during detection, resulting in a significant decrease in the resulting signals [69,70]. Charlermroj et al., in their 2022 study [71], developed an approach to detect live bacteria using an LFA (Figure 4). They conjugated AuNPs with bacteriophages and employed microarray technology to produce specific antibody fragments derived from anti-*Salmonella* bacteriophages. The test displayed specificity towards *Salmonella* Enteritidis, without showing reactivity to other bacterial strains, and was proved efficient, with an analysis time around 15 min and LOD of 1.0 × 10^7^ CFU.mL^−1^.

AMPs are short peptide fragments that play a key role in the immune system, providing the first line of defense against pathogens [72]. AMPs can bind to bacterial membranes through electrostatic and hydrophobic interactions. AMP-based biosensors can detect low cellular concentrations due to their high affinity for targeted bacteria. Their ease of synthesis and modification, inherent stability, and low cost make this type of biosensor promising for the detection of *Salmonella*. However, their mechanisms of action are not entirely clear, and they fail in recognizing *Salmonella* specifically, as they cannot differentiate it from other pathogenic bacteria [73]. In the study conducted by Kulagina et al., 2005 [74], the authors used biotin-labeled AMPs for the detection of *Salmonella* Typhimurium. The device performed well, with an LOD of 6.5 × 10^4^ CFU.mL^−1^.

### 4.2. Labels

A label or tag is a signal transducer, typically of a chemical or physical nature, that converts a biological response into an easily detectable outcome, resulting from the interaction between the analyte and the bioreceptor. The generated signal intensity is either directly or indirectly proportional to the analyte concentration [75]. In LFAs, commonly used labels include gold nanoparticles, magnetic nanoparticles, quantum dots, and enzymes, among others. It is essential for the properties of these materials to remain stable after their conjugation with biorecognition molecules. Although some labels can generate a direct signal, such as in LFAs using AuNPs, others may require additional steps to produce an analytical signal [76].

#### 4.2.1. Gold Nanoparticles (AuNPs)

**Figure 5:**
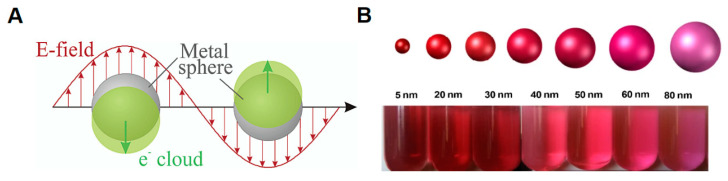
Optical properties of AuNPs. (**A**) Surface plasmon resonance of spherical AuNPs. Reprinted (adapted) with permission from [77]. Copyright (2003) American Chemical Society. (**B**) Optical property related to the size of AuNPs. Image reprinted (adapted) with permission from [78].

Gold nanoparticles are the most commonly used nanomaterials in LFAs. AuNPs display remarkable stability and a well-defined spherical shape, along with high affinity for proteins and biomolecules, favoring functionalization. AuNPs exhibit a surface plasmon resonance effect (Figure 5A), which contributes to excellent optical signaling, a feature directly linked to the sensitivity of LFA strips [77]. Signaling can be fine-tuned and enhanced through the deposition of silver and enzymes. The optical properties of gold nanoparticles rely on their shape and size. As the diameter increases (ranging from 15 to 150 nm), the wavelength of the absorption peak shifts towards longer wavelengths, leading to a darker coloration. With further size increment, the absorption peak tends to shift to even longer wavelengths, resulting in a solution coloration approaching dark purple (Figure 5B). Unlike spherical AuNPs, nanorods exhibit two absorption peaks: one in the visible range corresponding to the transverse plasmon, and the other near the infrared range corresponding to the longitudinal plasmon. Aggregation of AuNPs, which occurs when the distance between the nanoparticles is smaller than their diameter, causes a shift in the solution’s color from red to purple or blue. This color change is attributed to the coupling of surface plasmons, resulting in the absorption peak shifting to a longer wavelength [78].

According to the review by Wei et al., 2023 [79], LFAs have been developed using AuNPs as markers for the detection of different *Salmonella* serovars. The device proposed by Wu et al., 2021 [80], achieved an LOD of 4 × 10^5^ CFU.mL^−1^ in the detection of *Salmonella* Typhimurium, *Salmonella* Paratyphi B, and *Salmonella enterica*. Furthermore, LFAs based on AuNPs coated with citric acid combined with sodium ions—used to neutralize the surface charge and disperse aggregated AuNPs—resulted in a bluish-gray signal and a visual LOD of 10^3^ CFU.mL^−1^ [81]. An LFA enhanced with a signal based on the growth and accumulation of larger AuNPs was developed in 2018 by Bu et al. [82] for the detection of *Salmonella* Enteritidis, achieving a visual LOD of 10^4^ CFU.mL^−1^. In another study, Li et al., 2019 [83], immobilized multiple antibodies targeting different serogroups of *Salmonella* (O:2, O:3, O:4, O:7, and O:9) in the test line for target capture. The assay displayed high sensitivity and specificity for all five serogroups, with results read within 15 min.

**Figure 6:**
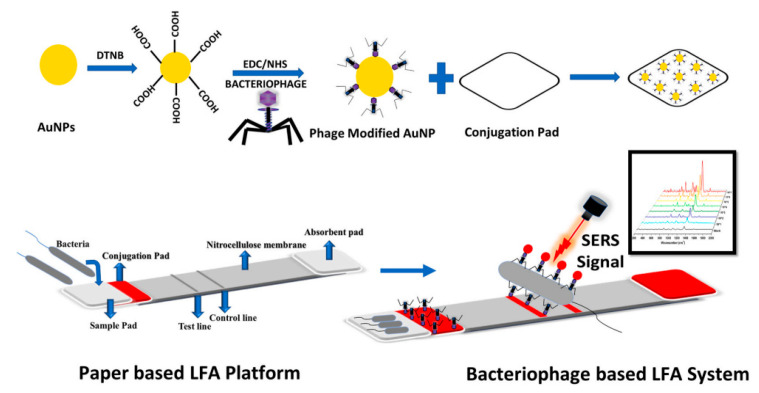
Illustration of the LFA strip using the SERS technique to compare the analytical performance of bacteriophages and antibodies in the detection of *Salmonella* Enteritidis. Reprinted with permission from [85].

AuNPs can also be coupled with the surface-enhanced Raman spectroscopy (SERS) technique, which involves an enhancement in the signal of inelastic scattering through molecules in the presence of a rough metal nanostructure [84]. Ilhan et al. [85] developed an LFA biosensor based on SERS using gold nanospheres labeled with Raman reporter molecules as probes. They compared the analytical performance of bacteriophages and antibodies for the detection of *Salmonella* Enteritidis. By combining SERS and colorimetric measurements for bacterial quantification, they achieved an LOD of 10 CFU.mL^−1^ and the method demonstrated good specificity (Figure 6).

#### 4.2.2. Magnetic Nanoparticle (MNPs)

Magnetic nanoparticles are currently being used in highly sensitive and quantitative biosensors through electronic detection systems with giant magnetoresistive (GMR) sensors [86]. MNPs include iron oxide nanoparticles, cobalt oxide nanoparticles, and nickel oxide nanoparticles. Among these, iron oxide nanoparticles are the most widely used owing to their biocompatibility, biodegradability, and superparamagnetic properties.

Some MNPs can produce a color in the test line that can be measured by an optical strip reader, and they can also serve as detection signals that are identified and recorded by a magnetic assay reader. When magnetic particles are on the nanoscale, their electrons are more confined and rotate in the same direction, resulting in a stronger localized magnetic field. Unlike macroscopic iron oxide molecules, iron oxide nanoparticles can be demagnetized when the external magnetic field is removed [87]. Due to these unique properties, certain MNPs approved by the FDA (Food and Drug Administration) are being employed as contrast agents in magnetic resonance imaging, as well as for cell isolation and the extraction of biological molecules. Wen et al., 2023 [88], used multifunctional Au-Fe_3_O_4_ nanoparticles in an LFA for the detection of *Salmonella* Typhimurium. In addition to the strip test, a photothermal test was also conducted using a portable infrared thermal camera to evaluate the results. This LFA achieved a visual LOD of 5.0 × 10^5^ CFU.mL^−1^ and a photothermal detection limit of 5.0 × 10^4^ CFU.mL^−1^.

**Figure 7:**
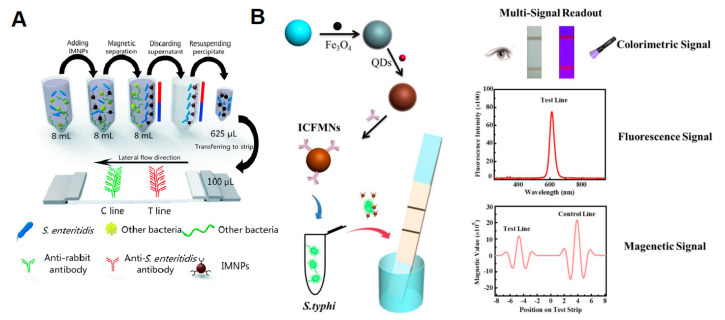
Magnetic nanomaterials as markers in LFAs. (**A**) Schematic diagram of an MNP-based LFA for the detection of *Salmonella* Enteritidis. Reprinted (adapted) with permission from [89]. (**B**) Scheme of the triple-signal LFA for the detection of *Salmonella* Typhimurium. Reprinted (adapted) with permission from [90]. Copyright (2018) American Chemical Society.

MNPs with optical and magnetic properties were used in an LFA engineered for the detection of *Salmonella* Enteritidis [89], as shown in Figure 6a. These MNPs were coated with antibodies and succeeded in reducing elution time and concentrating samples on a large scale, serving as both capture probes and signal reporters. The LFA thus developed showed a 1000-fold increase in sensitivity compared to the AuNPs-based LFA. In a study by Hu et al., 2019 [90], a colorimetric LFA based on fluorescent magnetic nanospheres was developed for the detection of *Salmonella* Typhimurium (Figure 7B). These nanospheres were produced using the layer-by-layer assembly technique, resulting in a functional fluorescent magnetic colorimetric probe. In this method, the antigen is captured and enriched by a multifunctional probe labeled with antibodies. The triple-signal LFA (colorimetric, fluorescence, and magnetic signals) exhibited high sensitivity and a wide linear detection range from 1.88 × 10^4^ to 1.88 × 10^7^ CFU.mL^−1^.

#### 4.2.3. Quantum Dots (QDs)

Also known as semiconductor quantum-confined fluorescent materials, QDs exhibit unique electrical and optical properties that are directly related to their size, allowing for tuning of fluorescence emission at different wavelengths. They are biocompatible and photochemically stable, with a broad and continuous absorption spectrum, allowing for efficient separation of excitation and emission wavelengths (commonly known as the Stokes shift) compared to organic dyes [91,92]. All these properties have led to the development of stable and efficient optical biosensors [93]. QDs enable detection by either enhancing or quenching the adsorption pathway, chelation, or direct interaction with specific conjugated bioreceptors or metal ions [94]. Their applicability, as well as that of their conjugated derivatives, has been reported in studies focusing on the development of fluorescence-based biosensors for pathogen detection and food safety [95,96]. Shang et al., 2021 [97], developed LFA strips labeled with nanobeads (QBs), combined with strand-displacement loop-mediated isothermal amplification (SD-LAMP) for the quantitative detection of *Salmonella* Typhimurium in food samples. These QBs rendered the test efficient and SD-LAMP enhanced the method’s specificity in the sandwich format. An LFA strip reader was used to measure fluorescence intensity, and the LOD obtained ranged from 10^2^ to 10^8^ CFU.mL^−1^.

**Figure 8:**
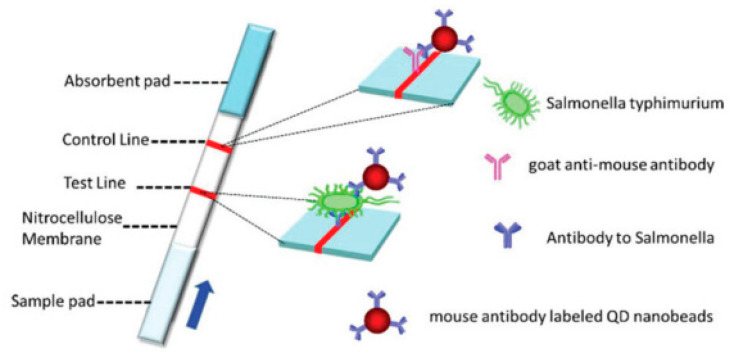
Illustration depicting an LFA strip manufactured and labeled with QDs for the detection of *Salmonella* Typhimurium. Reprinted with permission from [98].

In the studies conducted by Hu et al., 2020 [98], QD nanospheres were used as markers in an LFA strip for the detection of *Salmonella* Typhimurium. The LOD found was 5 × 10^3^ CFU.mL^−1^, and the test showed 100% accuracy, good specificity, and sensitivity (Figure 8).

## 5. Future Prospects of LFA as a Method for *Salmonella* Detection

LFAs offer several advantages over conventional methods and have become increasingly popular in the detection of foodborne pathogens, consistently advancing towards addressing food safety issues. They are rapid, low-cost, portable, sensitive, and specific assays, in addition to being user-friendly and easy to interpret. However, the sensitivity of many LFAs reported in the literature is insufficient to meet the regulatory limits required for detection. In most cases, conventional gold standard methods or PCR are used to complement LFAs in *Salmonella* detection. Considering the complexity of components in different food matrices, the analytical performance of LFAs is compromised when tested on real samples. An alternative to reduce interference and increase sensitivity would be the application of pre-treatment, separation, and concentration techniques that efficiently isolate, concentrate, and purify the *Salmonella* target [99]. Eliminating the requirement for sample enrichment would enable the detection of low concentrations of *Salmonella*. The results can be interpreted in a semi-quantitative or quantitative manner using strip readers or dedicated smartphone applications. These readers allow for quantitative analysis by converting color intensity into optical density values for the test and control lines [100]. To enhance sensitivity and specificity in the food supply chain, LFAs need to incorporate innovative technological strategies.

## 6. Challenges Related to LFAs in *Salmonella* Detection

According to regulatory standards that define microbiological criteria, *Salmonella* should not be present in ready-to-eat foods. Therefore, the development of highly sensitive detection methods is of utmost importance. Some LFAs manufactured and reported in the literature are still not sufficiently sensitive compared to gold standard and alternative methods. Moreover, the analytical performance of LFAs may be compromised when tested in enriched or real food samples due to potential interferences caused by more complex food matrices. Furthermore, detecting low concentrations of *Salmonella* through LFAs remains a challenge, particularly in real food samples. There are also concerns regarding the reliability of different methods in determining limits of detection for *Salmonella*, as some studies lack a comparison with established microbial testing methods [61], and higher detection ranges have been reported. Yet another challenge is the point-of-care application of LFAs, as it requires targeted sampling strategies for specific food matrices to detect *Salmonella,* not to mention the need for sample enrichment in culture media and incubation in temperature-controlled chambers prior to LFA analysis.

## 7. Conclusions

The use of LFA technology offers a compelling solution for meeting the requirements of quality and safety standards and enabling efficient and reliable detection and quantification of *Salmonella* in food. The high demand and market need for rapid and reliable pathogen detection are driving studies on this technique and contributing to improvements in its performance. In this review, the LFA has been presented as an alternative method to traditional approaches, offering portability, low cost, ease of use, and straightforward interpretation of results. Furthermore, it demonstrates high sensitivity, with the potential for even lower limits of detection depending on the combination of nanomaterials and bioreceptors employed. Overall, LFAs hold promise as powerful tools for the rapid and reliable detection of *Salmonella*, addressing the demands of the food industry and ensuring the safety and quality of food products. Continued research and development in this field will further enhance the performance and application of LFAs in the detection of foodborne pathogens.
